# When Collaboration Falters, Insensitivity to How Our Actions Affect Others Drives Inflated Self-evaluations

**DOI:** 10.1007/s42113-025-00250-y

**Published:** 2025-06-18

**Authors:** Michael Moutoussis, Meera Gosalia, Geert-Jan Will, Giles Story, Tobias U. Hauser, Aislinn Bowler, Siobhan Edinboro, theNSPNConsortium, Edward Bullmore, Raymond Dolan, Ian Goodyer, Peter Fonagy, Peter Jones, Michael Moutoussis, Tobias U. Hauser, Sharon Neufeld, Rafael Romero-Garcia, Michelle St Clair, Petra Vértes, Kirstie Whitaker, Barry Widmer, Gita Prabhu, Umar Toseeb, Junaid Bhatti, Laura Villis, Becky Inkster, Cinly Ooi, Pasco Fearon, John Suckling, Anne-Laura van Harmelen, Rogier Kievit, Ayesha Alrumaithi, Sarah Birt, Aislinn Bowler, Kalia Cleridou, Hina Dadabhoy, Emma Davies, Ashlyn Firkins, Sian Granville, Elizabeth Harding, Alexandra Hopkins, Daniel Isaacs, Janchai King, Danae Kokorikou, Christina Maurice, Cleo McIntosh, Jessica Memarzia, Harriet Mills, Ciara O’Donnell, Sara Pantaleone, Jenny Scott, Gita Prabhu, Raymond Dolan

**Affiliations:** 1 Functional Imaging Laboratory, Department of Imaging Neuroscience, University College London, London, UK; 2 Department of Clinical Psychology, Utrecht University, Utrecht, The Netherlands; 3 Max-Planck – UCL Centre for Computational Psychiatry and Ageing, Department of Imaging Neuroscience, University College London, London, UK; 4 Division of Psychiatry, University College London, London, W1 T7 NF UK; 5 Department of Psychiatry and Psychotherapy, Faculty of Medicine, University Tuebingen, Tübingen, Germany; 6 German Center for Mental Health (DZPG), Berlin, Germany; 7 Social, Genetic and Developmental Psychiatry Centre, King’s College London, London, UK

**Keywords:** Self-evaluation, Other-evaluation, Neuroeconomic task, Computational psychiatry, Self-serving bias

## Abstract

During high-stake interactions, people not only evaluate policies or outcomes, but also themselves and others. Such evaluations may be crucial for long-term outcomes, such as harmonious marriage, confident leadership and indeed mental health. Powerful evaluations occur during interactions, where people can support or let each other down. Thus, we implemented an interactive decision-making game, wherein two real-life participants explicitly evaluated themselves and their play-partner while playing an ecologically framed, probabilistic, iterated prisoner’s dilemma. To separate preferences from abilities, participants did not interact with the other directly, but instructed a computer avatar on how to play on their behalf. We tested a range of computational models of participants’ person-evaluations. In some, self-evaluation relied on regret or satisfaction regarding one’s decisions. However, the winning models relied directly on observed gains and losses. Here, evaluation of the self was proportional to how much one’s partner benefited, and vice versa. We found a marked self-positivity bias, which was most prominent in dyads where both partners often defected. Between participants, a self-positivity bias was explained by a reduced weight of one’s partner’s benefits onto self-evaluation. This suggests that the negative outcomes claimed to attract defensive, external attribution by attribution theorists are one’s partner’s poor outcomes. Further analysis suggested that a reduced sensitivity to others’ outcomes was associated with reduced earnings for the self, hinting at a functional role for person-evaluations in decision-making. Thus, we introduce a novel computational model that provides a concise account of self-serving bias in evaluations, as observed during risky dyadic interactions.

**Supplementary Information:**

The online version contains supplementary material available at 10.1007/s42113-025-00250-y.

## Introduction 

A positive assessment of the self is crucial for our well-being, and this is based on a holistic self-appraisal of our positive and negative ‘value’, alongside others’ perceptions of us (Elder et al., 2023; Taylor & Brown, 1994; Zhang et al., 2018). Evidence indicates that the brain computes moment-to-moment evaluations by integrating recent social outcomes, in a manner analogous to non-social emotions (Will et al., 2017). Finessing the ‘sociometer hypothesis’, we found that the rate of *change* in approval received from others matters more than the *average* level of approval in influencing self-esteem (Low et al., 2021). However, these studies, based on simulated social feedback, did not elucidate how evaluating self and other takes place in more real-life, interactive situations.

Social interaction studies based on game-theoretical paradigms have been popular with researchers, but may be perceived as rarefied (Colman, 2003; Rilling & Sanfey, 2011; Schilbach et al., 2013) and even deceptive (deceive-then-debrief practices), damaging ecological validity. In this study, we strove for ecological validity, introducing a new paradigm that entirely avoids deception, increases real-life vividness of exchanges and preserves the uncertain nature of real interactions. After validating this task, we went on to study the putative computations underlying self- and other-evaluation.

**Fig. 1:**
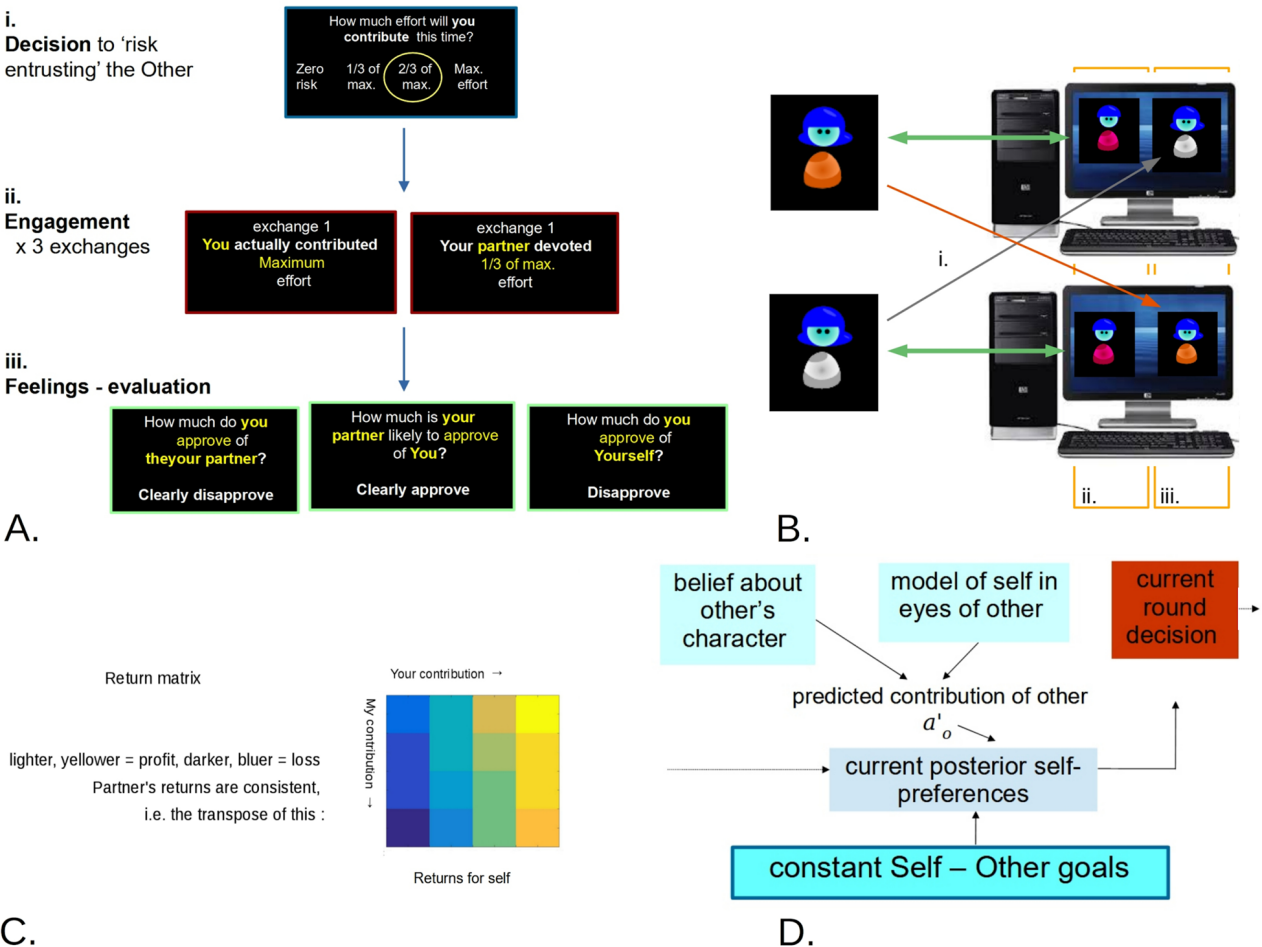
Task overview. **A** The three stages of each trial (total: 23 trials). (**i**) Participants decide how much to invest in during this trial, on average. The partner forms a similar decision, which is hidden. (**ii**) Noisy implementation of the decision taken in (**i**) for three rounds. (**iii**) The participant is asked how much they approve of themselves and the partner in this round, and also to mentalize how much the partner may approve of them. **B** Each participant does not play directly with their human partner, but does so via their avatar. (**i**) Before the task, each participant instructs their avatar about their preferred investment for each level of investment of the partner. (**ii**) During the scanning version of the task alone, participants first played with an avatar expressing averaged preferences of the previous experiments, (**iii**) then with the avatar trained by their real-life lab partner. **C** Returns matrix for the four levels of investment by each player. Temptation to free-ride is at top-right. **D** Markov decision generative model from the point of view of the Self, who holds self-other goals (bottom) and actively infers ‘who my actions would make me’ (grey-blue), according to which actions are taken in the current interaction (red)

We used a modified iterated prisoner’s dilemma game, with the addition that the outcome of each decision to risk social collaboration was followed by approval ratings of the self and of the other player. Additionally, participants rated how much the other player was likely to approve of them. On each trial, participants chose how much effort to invest in collaboration. Importantly, to increase ecological validity, the actual investment only noisily reflected intentions (Fig. 1A). Participants could choose among four intended levels of investment and were subject to the temptation to free-ride (Fig. 1B). To enhance ecological validity, two ‘partners’ met in the lab before and after the task, potentially exposing each other to feelings such as gratitude or embarrassment about how the task went. However, in our task, each participant did not interact with their real human partner directly. Instead, each participant interacted with an avatar simulating the behaviour of their specific real partner, thus affording a great degree of experimental control.

We build avatars whose decision-making approximated those of each participant. Each participant then played their partner’s avatar, and we could truthfully say that the avatar represented their partner’s beliefs and preferences. However, all avatars had the same cognitive abilities. Although we validated avatar behaviour, we stress that our study of self- and other- evaluation does not rely on actual accurate representation of a real partner’s behaviour (indeed it is standard practice not to use accurately individualized avatars, e.g. Moutoussis et al., 2016, Hula et al., 2021). To construct avatars, we first assessed each participant’s beliefs and preferences about self and others before the task started. This included what they believed others’ most prevalent preferences were, what their own preferred actions would be for each level of their partner’s actions, and measures of their certainty for each belief and preference. We inputted these self-reported preferences into avatars whose average behaviour was, as desired, substantially correlated with those of their ‘masters’. The decision-making model of avatars is illustrated in Fig. 1D and described in more detail below.

How are we to understand person-evaluation, including self-positivity bias, theoretically, and what computational models can clarify them? Other things being equal, people evaluate themselves more positively than others. Dominant attribution theories, and before them psychoanalytic theories, postulated that people do this simply in order to feel better—a self-serving bias (Robins & Beer, 2001; Wang et al., 2017). Such a view is teleological or even circular; but from a computational point of view, we can clarify it by hypothesizing that self- and other- evaluation follow different rules, and report different outcomes of the interaction that may be useful for decision-making. Models linking self-evaluation to optimal decisions are then useful, as they provide a normative basis for studying social evaluations. Here, we hypothesized that we evaluate others depending on how they deviate from our ideals, whereas the self is primarily judged by how good our behaviour is *conditional* on others’ character. For example, we may approve of ourselves investing little with unhelpful, uncooperative people. This class of models would provide a normative theoretical rationale, of *why* a self-positivity bias is observed, but must also explain *what* the map of features of interactions onto evaluations is.

Reinforcement learning may be defined in different ways, but here, let’s focus on its core assumption that a person knows what they strive for and what they avoid, i.e. that ‘rewards’ and ‘punishments’ are well defined in terms of outcomes. A key theoretical advantage of reinforcement-learning models is that they can provide powerful constraints for further theory: Precisely describing *what* the map in question is powerfully constrains the ‘why’ (and the neural ‘how’, of course) of subsequent theory. We thus compared and contrasted belief-based models with returns-based (‘reinforcement’) models based on previous, descriptively successful, but less normative reward-prediction-error based models (Low et al., 2021; Will et al., 2017).

Here, we hypothesized that social approval of self and other during interactions would be interlinked, depending on material outcomes for the self, but also be informed by those of one’s partner (Fehr & Camerer, 2007; Fehr & Schmidt, 1999; Hula et al., 2018). At its simplest, this motivates selection of linear maps from vectorial outcomes (over self and other) to vectorial evaluations (again, over self and others) as reinforcement learning models.

## Methods and Materials

### Participants

The present studies were approved by the University College London Ethics Committee (3450/002). All participants were recruited from the healthy adult volunteers of the ‘Neuroscience in Psychiatry Network Project’ (NSPN) cohort. They were healthy 18–25 year old people recruited in either North London or Cambridge, UK, representative of the UK as a whole in terms of gender and ethnicity (Kiddle et al., 2018).

Our pilot study (Study 1) included 24 participants. To determine the minimum sample size for the main study, we calculated the number needed for the difference in evaluations between self and other ('self-positivity') to reach conventional statistical significance (*p* < 0.05) if the Minimum Effect of Interest was to be half the mean difference found in the pilot study (see Supplement & Fig. S1). This gave a minimum of *N* = 41. We actually recruited a total of 64 participants for the main study (study 2) reported here. Table 1 gives their basic characteristics.

**Table 1:** Makeup of the recruited samples in the pilot and main studies

	Study 1 (pilot)	Study 2 (main)
Age	18–25	18–25 M = 22.75, SD = 4.85
Total sample size	24	64
Self-reported female	12	29
Self-reported male	12	35
Self-reported other gender and/or no answer	0	0
Performed task in the lab, outside the MRI scanner	24	24
Performed task in the lab, inside the MRI scanner	0	40

In the main study, the only other inclusion criteria were suitability for fMRI scanning (not reported here). On 16 occasions, the partner of a scanned participant did not attend, and an approximately age-matched, female research assistant filled in, but their data was not analyzed. All participants gave written informed consent and were paid for their time. Hourly rates were specifically instructed by the Ethics Committee, as the overall NSPN study also included younger adolescents, who were not sampled here.

Separately, participants were paid for their returns in the game. They were truthfully told that the winnings and losses, in play-points, of themselves playing their partner’s avatar, and of their avatar playing their partner, would be totalled and converted to money which if they did well would be in the region of 5 pounds (this was the approximate average of the pilot group). They were not told what would happen if they incurred net losses; in reality, net losses were converted to zero pounds.

### Experimental Task

This is shown in Fig. 1 and was based on a probabilistic, four-level iterated prisoner’s dilemma. Prior to the experiment, participants were extensively instructed and explicitly asked about different scenarios, including ‘what do you think your partner will do if you let them down?’ so that they understood the possible consequences of honoring cooperation, letting the other down, etc. They knew their own decision, but had to infer their partner’s decision from noisy outcomes. The computer played three ‘rounds’ based on the decisions of the two players. Having been trained on the returns matrix (Fig. 1C), participants were only shown the contributions of the two players, not directly their returns. After each triad of ‘rounds’, participants were asked to give self-approval ratings, approval of their partner and (in the validation samples only) their guess as to how much their partner approved of them. The game ended pseudo-randomly, to reduce end-anticipation effects.

### Avatar Players

Participants did not play each other directly, but each played with an avatar representing the other participant. Thus, participant 1 would play with participant 2’s avatar, while participant 2 would play with Participant 1’s avatar (Fig. 1B). Avatars were powered by a partially observable Markov decision process (Fig. 1D), which instantiated principles of realistic, limited human rationality. First, agents had a fixed depth of theory-of-mind: ‘I believe that you believe that I am the kind of person that prefers to do X’. This, was, however, modulated by rich prior beliefs, which described ones’ own preferences, but also those of others (Moutoussis et al., 2014). This was mathematically motivated by the finding that in game-theoretical tasks, a simple a priori strategy can exactly substitute, in specific contexts, for decisions that maximize returns through sophisticated (and computationally very demanding) recursive mentalizing (Yoshida et al., 2008). To illustrate, it may be possible but difficult to estimate the long-term expected costs of lying in a specific community and hence decide that telling the truth is the best decision—or one may follow a simple moral imperative, to the same effect. This makes models more easily interpretable in terms of psychology and phenomenology (‘algorithmic’ Marrian models, (Marr, 1982), complementing the ‘ideal computational’ models of our previous work (Hula et al., 2021; Moutoussis et al., 2011).

Structured beliefs about self and other preferences are not only copmutationally practical, but provide potential insights into psychology. People—and here, their avatars—act to ‘make themselves who they want to be’ within their relationships, thus ‘actively inferring’ about themselves. This is an Active Inference approach to interpersonal settings (Moutoussis et al., 2014). Each agent has goals (*C*_*s*_) not only about themselves, but about the relationship they enter, i.e. joint goals about self and other. Goals are then prior probability distributions (Friston et al., 2012; Moutoussis et al., 2014; Smith et al., 2022) over the joint actions of self and other, where higher probability is assigned to more desirable action combinations. A desired distribution over dispositions of both self (S) and others (O) can hence be expressed as probabilities, or dispositions, to act:

1 $${C}_{S}:={P}_{taste S}={P}_{goal S}\left(S,O\right)=P\left(S\left|O\right.\right)P\left(O\right)$$

Here, the conditional distribution *P*(*S*|*O*) specifies the desired disposition of the self to act, given the other’s disposition. Experimentally, we asked participants what their own favorite action would be, for each of the four possible actions of their partner, informing P(*S*|*O*). Then, we asked for their partner’s ‘ideal action’ for them, providing the mode of P(*O*). Then, we asked them how much they would like a person preferring actions one step less cooperative than their ideal, and used this to estimate the entire P(O), approximating it by a discretized beta distribution.

In the decision-making model, agents assumed that others have different goals, *C*_*O*_, which they also try to fulfill. Agents were hence motivated to infer their partner’s *C*_*O*_. They did this based on their observations, taking into account who their partner may have inferred them to be. For example, ‘they have witnessed me acting erratically’. They then act to provide evidence steering joint outcomes to diverge as little from *C*_*S*_ as possible. In our example, ‘my partner will invest more in me if my actions provide evidence against being my erratic’. This, then, implements a minimum set of principles of risk-sensitive active inference (Friston et al., 2012).

Prior to the main game play, each participant performed a practice task, playing with a ‘Ms Average’ avatar, which had modal preferences from a pilot run. This game play—not analyzed here—prepared them for play with their real-life lab-partner’s avatar. It included reminders of the total returns on each trial, enhancing participants’ training on the relationship between joint actions and outcomes. Using avatars meant that there were as many dyads as players.

### Decision-Making Modelling

In the rest of this section, we provide details of the decision-making model for the interested reader. They are not essential to understand this paper’s hypotheses and findings. So, to make the best possible decision in the light of a Theory of Mind of ‘I believe that you believe that I am the kind of person that prefers to do X’, an agent (say the Self, *S*) first updates their approximate propensity for decisions, *d*_*S*_, in the eyes of the partner, *p*(*d*_*S*_) i.e. their current persona:

2 $${p}_{persona S}\left({d}_{S};t-1\right)\propto p\left({a}_{s}\left(t-2\right)\left|{d}_{s}\right.\right){p}_{persona S}\left({d}_{S};t-2\right)$$

where *a*_*S*_ are actions of the self, t-1 is the time when the last action was witnessed and t-2 the one before that. At the same time, *S* updates their beliefs about *C*_*O*_. For each possible, discretized state *i* of *C*_*O*_:

3 $$p\left({C}_{o}^{i};t\right)\propto p\left({a}_{o}\left(t-1\right)\left|{d}_{o}^{i}\right.\right)p\left({C}_{o}^{i};t-1\right)$$

We assume that agents keep track of this distribution, but use only its a posteriori* maximum* for downstream decision-making. So *S* can now expect *O* to make decisions so as to best fulfills their own goal preferences:

4 $${d}_{o}'=\underset{{d}_{o}}{\mathrm{argmin}}{D}_{KL}\left[p\left({a}_{s}|{d}_{s}'\right)p\left({a}_{o}|{d}_{o}\right)\Vert {C}_{o}^{MAP}\right]$$

where the dash denotes estimated, rather than observed, decisions. Finally, S makes a decision on the basis of a simple softmax function of the cost of that decision, in terms of divergence from my preferred goals:

5 $$\pi \left({d}_{s}\right)\propto exp\left(-{D}_{KL}\left[p\left({a}_{s}|{d}_{s}\right)p\left({a}_{o}|{d}_{o}'\right)\Vert {C}_{s}\right]/\tau \right)$$

### Belief-Based Evaluation Modeling

Here, we hypothesized that people evaluate their partner on the basis of how well their decision-making matched that of their ideal partner. Specifically, the probability of the actions taken by the partner under this ‘ideal partner’ policy was calculated, forming an evaluation score. A ‘partner positivity bias’ parameter was also introduced, which systematically shifted reports towards a higher or lower value. This modeled the fact that people may adopt or at least report biased evaluations compared to their initial inferences, for example to feel better about themselves or to please experimenters. A further partner-evaluation-noise parameter influenced the evaluation score reported (Supplement Equations S7 – S8). The (unseen but guessed) evaluation of self by the partner should then follow the model of other evaluation, but applied to the probability of self-actions under the inferred preferences of the partner.

Approval of the self was again based on the probability of the actions one actually took under ‘ideal’ decision-making, but followed a ‘regret model’ (Coricelli et al., 2007; Steiner & Redish, 2014). That is, the ideal reference policy was the one that *would* obtain *had* the participant known their partner’s actions in this trial. We thus hypothesized that the self was evaluated against a different criterion, relevant to the ideal policy that one might follow, rather than one obtaining unconditionally. Once this probability was calculated, it was mapped to evaluations reported following the same procedure, but with independent self-positivity bias and self-evaluation-noise parameters. Under a hypothesis that the different rules used for evaluation explained self-positivity bias, then this predicts that the two positivity bias parameters, for other and self, should not differ.

### Reinforcement-Learning Evaluation Modeling

Drawing on previous modeling of self-evaluation and emotion (Emanuel & Eldar, 2023; Rutledge et al., 2014), we hypothesized that current evaluations should depend on a weighted sum of both most recent and past outcomes for self and other, with greater weights for more recent outcomes. If updated at each trial, this weighted sum is estimated by a simple iterative learning rule (Emanuel & Eldar, 2023; Low et al., 2021). Note that if iteratively estimated, a weighted sum of past prediction errors reduces to a (somewhat differently weighted) sum of past outcomes, as each past expectation is itself a weighted sum of past outcomes. Next, we note that evaluation of one person may depend on the gains or losses of the other; thus, rather than two one-dimensional equations, we should allow for a matrix form:

6 $${\overrightarrow{E}}^{t}={\overrightarrow{E}}_{0}+{W}_{x}{\overrightarrow{E}}^{t-1}+{W}_{r}{\overrightarrow{R}}^{t}$$

Here, *E* is a vector of self- and other- Evaluation, *R* is a function of outcomes (say, returns and prediction errors), *W* are constant matrices and *E*_0_ encodes intercepts for self and other.

## Results

### People Can ‘Teach’ Their Avatars to Invest in Others or Not

**Fig. 2:**
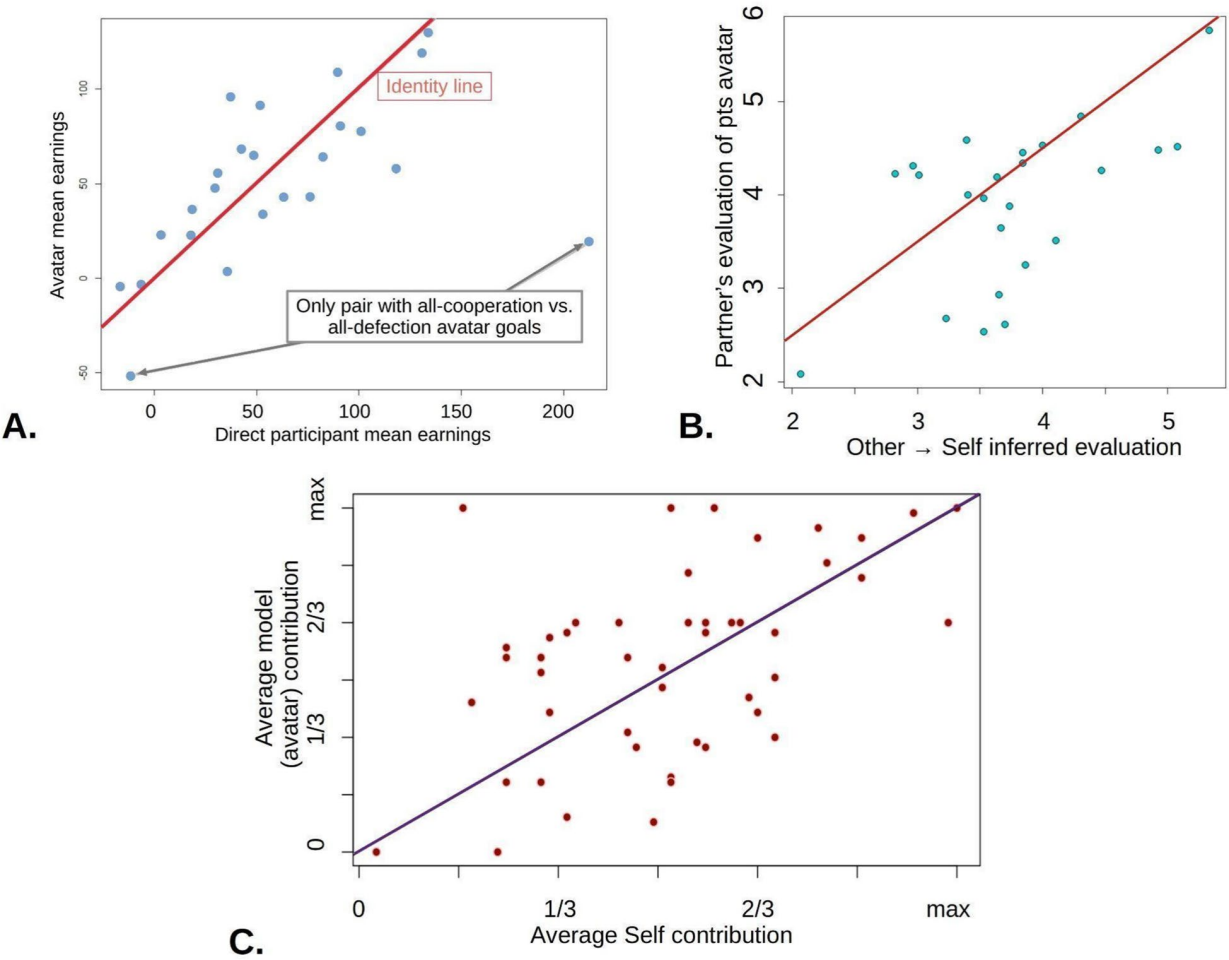
Avatars behaved similarly to the participants that instructed them, and even elicited similar evaluations. **A** In study 1 (*N* = 24), avatar earnings were very similar to the corresponding participant (Spearman rho = 0.53, *p* = 0.008 including one outlier pair). **B** The average guess of a play-partner’s evaluation of the participant correlated with the actual average evaluation that the real-life lab partner made of the participant’s avatar. The guess, or ‘mentalizing the self’ questions were only used in study 1 (rho = 0.46, *p* = 0.022). **C** The main study (*N* = 63) replicated the decision-making results. Average self-contribution correlated to avatar contribution, Pearson *r* = 0.50, *p* = 0.0003)

Study 1 showed that the behaviour of participants was similar to that of the avatars that they instructed, supporting the hypothesis that people could disclose their two-person goals to instruct their avatars. It also showed that given these instructions, the model driving the avatars resulted in overall decision-making similar to their instructors, and that feelings of approval elicited by this behaviour were also well aligned (Fig. 2A, B). This was replicated in our main study (Fig. 2C), supporting the validity of further analyses.

### Lack of Collaboration Is Associated with Self-serving Evaluation Bias

In study 1 (pilot), participants evaluated themselves more positively than their partners, mean difference = 0.76, *t*-test *p* = 0.00086. We used this for the power calculation of the main study. We asked the sample size that would be required to detect a Minimum Effect of Interest of half this mean difference, assuming a t distribution, giving in *N* = 41. (Online power sample size calculator: www.gigacalculator.com). However, we recruited *N* = 70 for study 2 (main study) so that, allowing for attrition, there would be at least 30 participants in the brain-scanning half of the sample (not analyzed here).

**Fig. 3:**
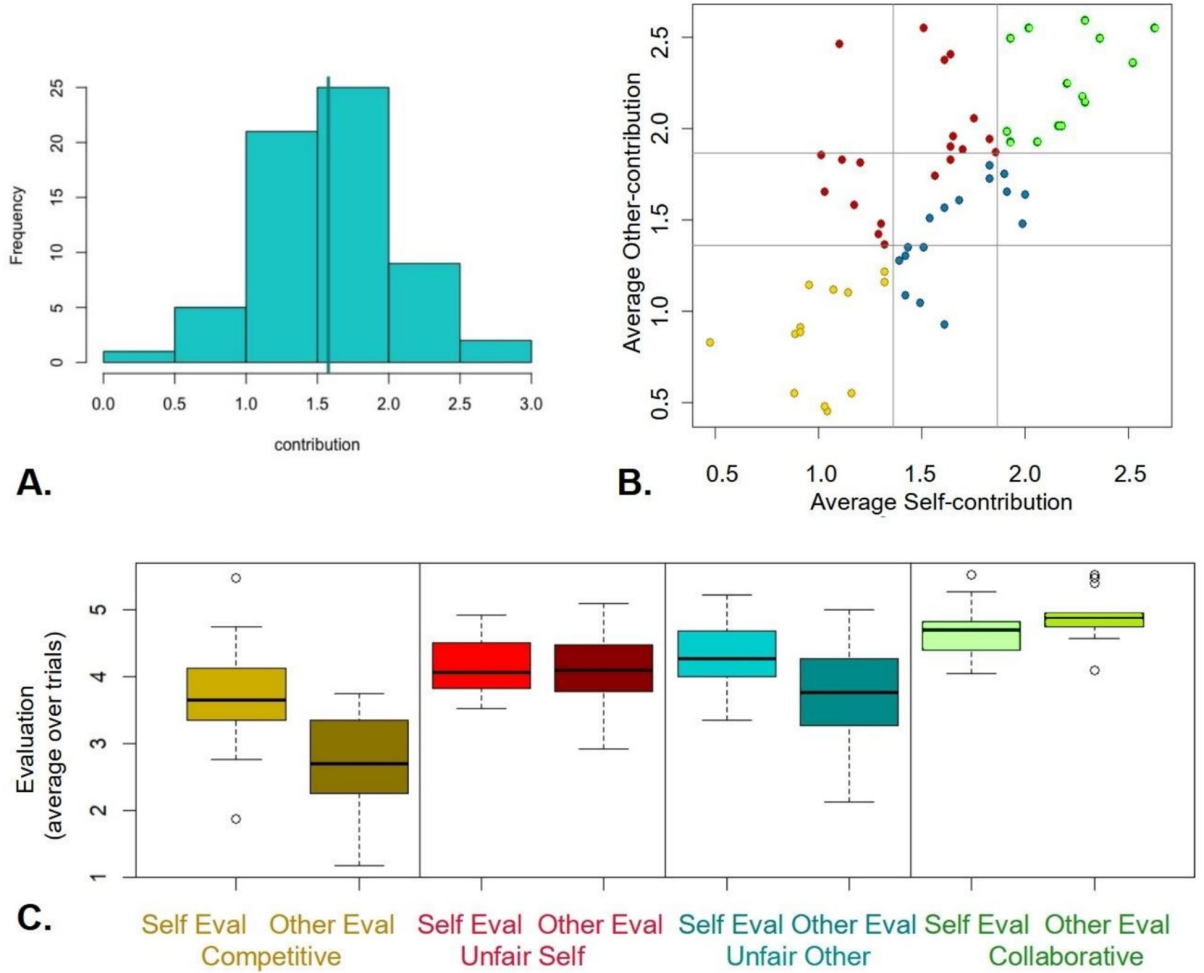
**A** Distribution of average participant work contributions (main study). Despite training, the mean (green line) is just into the cooperative zone (> 1.5). **B** Dyads are separated with respect to the average level of cooperation into mutually competitive (both partners contributing in bottom tertile, lower left area, gold), mutually cooperative (both in upper tertile, upper right, green), unfair self (not in the first two, plus self-contribution < partner contribution, upper left, red) and unfair other (the rest, lower right, cyan). **C** Self- and other- average evaluations for each group. Note the marked self-positivity bias in the competitive group, and absence of low self-evaluation in the ‘unfair self’ group

Main study participants approved more of themselves than of their partners, showing self-positivity biases (Fig. 1C) and replicating study 1 (*p* = 0.0057, mean difference = 0.33, CI = 0.10, 0.562)). They showed more cooperative than uncooperative behaviour, but only marginally so (Fig. 1A). Self-positivity was structured: Uncooperative behaviour by the partner tended to reduce other-approvals (as might be expected), but even unilateral uncooperative behaviour by the self did not reduce approvals of the self below that of the partner (Fig. 3C, red). Strikingly, the most pronounced self-positivity bias was observed when neither partner played cooperatively (one-way ANOVA of average self-other evaluation difference vs. cooperation group *p* = 0.00117. Tuckey’s test: competitive > collaborative *p* = 0.00152, competitive > unfair self *p* = 0.0114, unfair other > cooperative *p* = 0.0653, all other pairwise comparisons *p* > 0.32).

### Reinforcement-Learning Models Performed Better Than Beliefs-About-Decisions Models

**Fig. 4:**
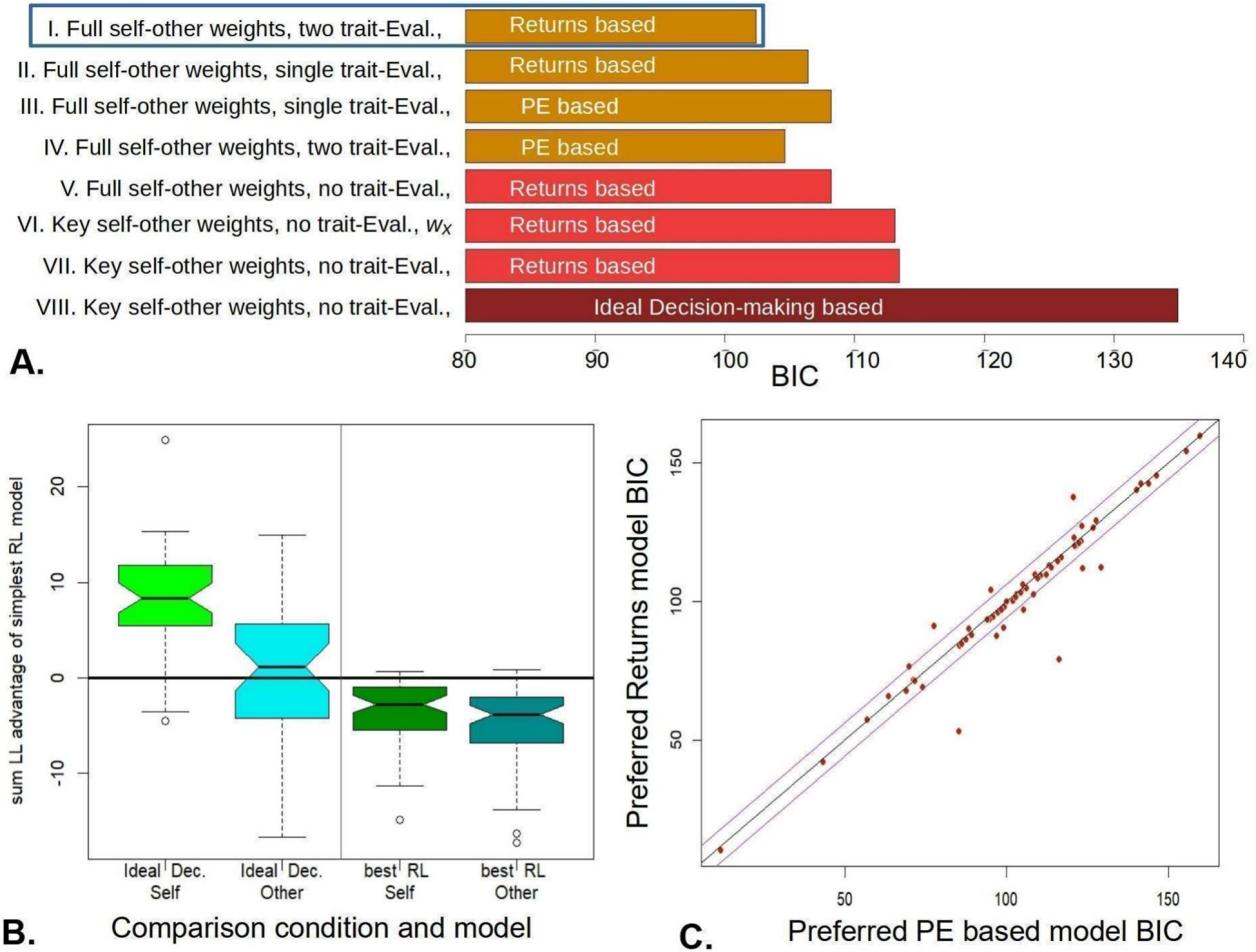
Key model comparisons **A** Median BICs for key models. The returns-based, full-self-other-weights, full trait-Evaluation, returns-based model (I) did best. **B** Model VII taken as reference, and its advantage over the ‘Ideal Bayesian decisions’ model for self and other (VIII) is shown at left; and (dis)advantage compared to more complex, winning model I at right (uncorr. Wilcox. p for the latter = 3.7E-4). Notch is the approximate 95% confidence interval for the mean. **C** The preferred Returns-based model (I) is more accurate than the best PE-based model (IV) for a few subjects, but for most, it gives a very similar account of the data with a lesser complexity penalty (uncorr. Wilcox. *p* = 0.0069)

The ideal decision-making evaluation model, which operationalized our primary hypothesis, relied on beliefs about decisions. For most participants, other-evaluation amounted to how far the other’s behaviour deviated from a highly cooperative one. This model performed better than chance, but also better than the self-evaluation ‘comparison with ideal decision’ belief models (Fig. 4B, left; Wilcoxon *p* < 1e-5 for log-likelihoods of self vs. other evaluation sub-model). This provided evidence against our hypothesis that Self-evaluation is primarily based on approval (or regret) about one’s own decisions.

We therefore formulated simple reinforcement-learning (RL) model, as per Eq. (6), which relied on outcomes rather than actions, using a weighted-average of past outcomes, as reported in our previous work of self-esteem in non-interactive contexts (Will et al., 2017). Even the simplest RL model, which only contained parameters 2, 3, 7 and 8 of Table 1, gave a much-improved fit (Wilcoxon *p* < 1E-05 for individual level BIC values in favour of model VII vs. VIII in Fig. 3A). Finally, we refined the RL model by stepwise introduction of the terms, and corresponding parameters of Table 2. The winning model included a full self-other weights matrix and a full baseline evaluation vector (Fig. 4), but we note that this fine-tuning of the RL model was exploratory and subject to future replication.

**Table 2:** Summary of parameters of reinforcement learning evaluation models

	Parameter name	Symbol	Explanation
	Winning model parameters		
1	Weight of self-returns on self-eval	*w*_*SS*_	1–4 are the elements of a matrix that transforms the return-related observations into an update of self- and other- evaluations
2	Weight of other-returns on self-eval	*w*_*SO*_	See 1. above
3	Weight of self-ret. on other-eval	*w*_*OS*_	See 1. above
4	Weight of other-ret. on other-Eval	*w*_*OO*_	See 1. above
5	Trait self-evaluation	*E*^*0*^_*Self*_	Additive ‘trait’ evaluation of self-induced in the context in question, but independent of interaction returns
6	Trait other-evaluation	*E*^*0*^_*Other*_	As for 11, but for ‘trait’ evaluation of other
7	Autoregression weight	*η*	Weight of learning from new outcomes, relative to previous person-evaluations
8	Decision noise	*σ*	A higher decision-noise parameter means more stochastic decisions; however, this can be overcome through learning strong action-values
	Parameters explored, but not included in preferred model (see supplement)		
9	Expectation value learning rate	*λ*_1_	Learning rate in PE-based models, by which participants update expectations about returns for self and other
10	Prediction-error (relative to outcome) learning rate	*λ*_2_	Relative weight of PEs vs. outcomes on return component
11	Cross-autoregression weight	*w*_*Ex*_	Weight allowing for self- and other- past evaluations to influence other and self-current values
12	Decision noise floor	*ζ*	Motivation-independent noise

### Prediction Errors Not Needed for Self- and Other-evaluation

As expectations are calculated from weighted sums of returns, a diminishing power series sum of prediction errors (PEs) is necessarily a diminishing power series sum of returns, albeit with different weights. Whereas PEs may be used for computations in the brain, at the algorithmic level simply using returns provided a more parsimonious account of the data (compared to using PEs or mixtures of PEs and returns in the outcome-dependent term of Eq. 2). In any case, the winning models (Fig. 4A) contained a full matrix of weights by which self- and other- returns updated both self- and other-evaluations (*W*_r_ in Eq. 2). They also contained separate trait-evaluations for self and other. The resulting reinforcement learning models captured precisely self- and other- evaluations, and also their difference, that is, the self-positivity bias. To quantify this, we first generated synthetic data using the fitted parameters, using veridical experimental returns. For each participant, we formed the average of the first 6, middle 11 and last 6 trials. The respective adjusted *r*^2^ were 0.88, 0.94 and 0.86 respectively, all *p* < 1E-10. Averaging over all trials, the self-positivity score (average self-eval.—other-eval) was almost perfectly captured, adjusted *r*^2^ = 0.99. Note that the model could do this last fit in an un-interesting manner, by simply capturing the average behaviour in the ‘trait evaluation parameters’ (5 and 6 in Table 1). This was not the case.

### Self-positivity Bias Decreases with Increasing Weight of Other-Returns on Self-evaluation

**Fig. 5:**
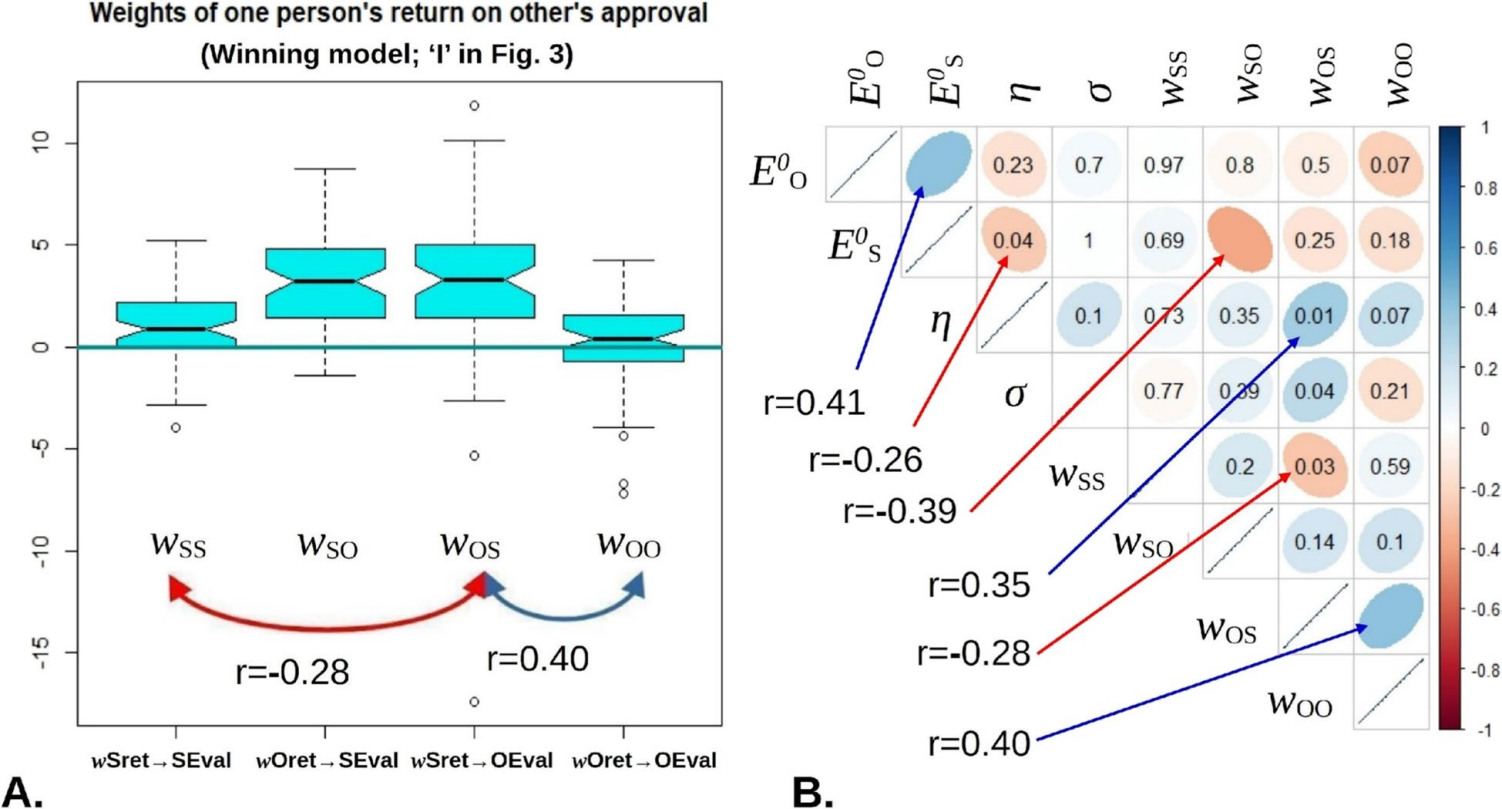
How parameters are distributed in the sample **A** Other-Evaluation is most strongly influenced by self-returns (w_OS_) and vice versa (w_SO_). The more one relies on other-returns for self-evaluation, the less they rely on self-returns for the same (red), and the more strongly their evaluation of the other is influenced by that other’s returns (blue). **B** Full correlation structure between posterior parameter estimates, with bivariate correlations pointed at for Bonferroni-corrected significant values

We then regressed the self-positivity bias against all the parameters. The weight of other-return on self-evaluation parameter was strongly associated with self-positivity (beta = − 0.20, *p* = 2.3E-5). The weight of other-return on self-evaluation parameter was also significantly, but much less strongly associated (beta = 0.068, *p* = 0.035). Other parameters, notably trait evaluations, did not contribute to self-positivity. The two parameters that did were the weights that most heavily influenced evaluation updates (Fig. 5A). Linear regression with parameters as predictors was highly significant, but accounted for a far smaller amount of variance than the synthetic data (adjusted *r*^2^ = 0.36, *p* = 4.6E-5).

We then explored the correlation matrix between all parameters, where this pointed to a number of psychologically interesting results. People who had high trait self-evaluation also tended to have high trait other-evaluation (Fig. 5A, B); while those whose self-evaluation was most strongly influenced by their partner’s gains were also more influenced by the same outcome to form other-evaluations (Fig. 4B).

We asked whether the association between self-positivity and *w*_*SO*_ was mechanistic, or due to a confounder (e.g. the structure of returns for those with high or low *w*_*SO*_). To test this, we sampled scrambled *w*_*SO*_ and again created synthetic data. This time, the overall sample did not show a statistically significant self-positivity (paired *t*-test *t*-value = 0.82, *p* = 0.414), while the simulated self-positivity only explained adjusted *r*^2^ = 0.54 of the experimentally obtained values (*p* < 1E-10). These results indicate that *w*_*SO*_ is mechanistically important for self-positivity bias.

Of the other parameters, we note that only ${~}^{1}\!\left/ \!{~}_{3}\right.$ of our sample was fitted with autoregression weights less than 0.9 (*η* in Table 1; first tertile = 0.894, median = 0.98). As each evaluation followed three outcomes played noisily by the computer on the basis of the last decision, this means that most participants made evaluations solely on the basis of these last three outcomes. Although our study was not optimized to study the impact of evaluative style on outcomes, we performed a preliminary analysis regressing average returns for the self of evaluation parameters. The regression was significant, *p* = 0.011, adjusted *r*^2^ = 0.185. Two parameters had significant, positive weights: *w*_*SO*_ (*t* = 2.51, *p* = 0.015) and *E*^*0*^_*Self*_ (*t* = 2.38, *p* = 0.021).

## Discussion

Starting from a premise that self- and other- evaluations are interlinked and clinically important (Udachina et al., 2012, 2017), we examined evaluations during meaningful interaction and found that evaluations depended on both self and other outcomes, with self-positivity bias crucially depending on how benefits to one’s partner weighed into self-evaluation. We utilized a task played by pairs of participants, where each instructed an artificial intelligence (AI) avatar as to their preferences for collaboration and competition, and each participant then played with the other’s avatar. This ensured good experimental control and realistic motives. Trial-by-trial self- and other-evaluations showed a characteristic pattern of self-positivity, where self-evaluations were on average greater than other-evaluations. Computational models first explored whether beliefs about how good one’s decision-making was, and how close to the ‘ideal’ one’s partner was, accounted for evaluations. We then explored reinforcement-learning models, where evaluations depended on outcomes, rather than beliefs about actions. Reinforcement-learning models performed much better, and provided evidence that the lower the weight of one’s partner outcomes on self-evaluation, the greater the self-positivity bias.

A key methodological advance was the use of preferences about relationships of real-life participants, to inform the behaviour of avatars playing on their behalf. In this way, we personalized the ‘bots’ used in previous studies (Hula et al., 2018). We also used salient returns that have both a psychologically immersive (‘he let me down’) and monetary value. The fact that people’s actual behaviour correlated with that of their avatars indicated that (a) they could and did self-disclose their preferences and (b) that the generative model (the AI) implemented these successfully, on average. The procedure guaranteed to participants that their computer partners were of fixed and average intelligence, and that variation in behaviour was either due to chance, or to motivation. We found preliminary evidence that participants playing the other’s avatar (A playing B’s avatar) estimated how the partner (actual B) would evaluate them (A), similar to the evaluation that their partner (B) actually made of their own avatar (A’s avatar), showing remarkable theory-of-mind.

Self-evaluations were higher than other-evaluations, as expected from a literature on self-positivity biases. This was significant in the overall sample and was particularly pronounced if both participant and partner played uncooperatively.

Strikingly, mean self-evaluation was almost identical to other-evaluation when the self-behaved less cooperatively than the partner. Thus, we speculate that the ‘external-personal attribution for negative events’, whose function was once hypothesized to be to make people feel better (Murphy et al., 2018), is something healthy people do to avoid negative evaluation of their own unfriendly actions during interactions. It is outside the scope of this report to ascertain whether the benefit of this is intrinsic (psychological defence) and/or social (to avoid opprobrium or material punishment by the social milieu).

When modeling self-evaluations, we found that models explaining personal evaluations in terms of the gains and losses of self and others out-performed those that compared actions taken to the best possible ones. Crucially, people did not primarily approve of themselves the better they did in task, but the better their partner did—and vice versa. This contrasts with a Fehr-Schmidt model-based hypothesis, which might place most weight for self-approval on self-returns (Fehr & Schmidt, 1999). People did not evaluate themselves qua *homines oeconomici* (Crockett et al., 2014). Remarkably, baseline or trait-evaluations of self and other did not contribute to self-positivity bias.

Winning models estimated evaluations as based on a weighted sum of outcomes, with rapidly decreasing weights for outcomes further into the past, rather like self-esteem models in non-interactive contexts (Low et al., 2021; Will et al., 2020). Winning models did not sum over prediction errors, unlike previous work, but simply over returns. We note, however, that if the weights of a PE-based model are allowed to vary freely, this closely approximates a returns-based model. Thus, the superiority of returns-based models points more to their algorithmic parsimony, rather than whether PEs or returns are used at the implementation level in the brain for the present purposes (Marr, 1982).

Most importantly, one parameter alone correlated with self-positivity bias, namely the weight of returns for one’s partner on self-evaluation. Insensitivity to others’ returns increased self-serving bias, and when this parameter was shuffled across participants in synthetic data, overall self-serving bias was no longer significant. This is interesting, as self-serving bias was mostly found in mutually competitive interactions, meaning that if a negative outcome is psycho-socially defended against, it is specifically the poor returns for the partner. Though only this parameter linearly correlated with self-positivity bias, we note that evaluation parameters showed complex patterns of correlations between them, even under strict Bonferroni correction for multiple comparisons (Fig. 5). Some of these correlations are understandable in psychological terms—for example, people who rely more on trait evaluations for the self, also do so for their partner. We caution that it would be unwise to interpret this pattern in detail prior to its replication. Preliminary analyses indicated that a greater sensitivity to others’ returns for self-evaluation also predicted a greater average return for the self.

Arguably the most important limitation of our study is that our key findings emerged from model exploration after our initial normative hypothesis was relatively unsuccessful (i.e. that people would evaluate themselves based on how close their most recent decisions were to their ideal decisions, a ‘regret model’). Thus, the superiority of our matrix model based on self- and other- returns needs further validation and theoretical understanding. Another limitation is that the framing of each trial may have encouraged participants to perceive it mostly as self-contained, as evidenced by the high values of parameter *η*. Thus, the task was not designed to assess the trial-to-trial impact of person-evaluations on decision-making. This should be the object of further research, taking into account that people may interact in part so that they approve of themselves and others in future, a form of affective forecasting (Charpentier et al., 2016).

The paradigm introduced here offers rich avenues for further research, including understanding self- and other- evaluation in relational psychopathology. This is central to psychiatric disorders ranging from paranoia to post-traumatic and personality disorders (Luyten et al., 2020; Moutoussis et al., 2013; Zavlis, 2023). It would be important to understand how developmental exposure or adoption of strictness (Garcia et al., 2020), and clinical measures such as paranoia (Barnby et al., 2022) affect self- and other- evaluation dynamics, possibly through preferences about how ideal partners behave. Future research can also take forward the current advances in combining ecological validity with experimental control, especially providing holistic sensory feedback, as facial expressions and experience of the body are crucial for social interactions (Hamilton and Holler, 2023; Zhang et al., 2021).

In conclusion, we provide behavioural and computational evidence that during social interactions which may be of mutual benefit, but where participants may also let each other down or exploit each other, being sensitive to the benefits and costs of one’s partner is more important than other considerations. A decreased sensitivity is associated with self-positivity bias, which is itself prominent when mutually un-cooperative interactions occur, consistent with attribution theory. On the other hand, a higher sensitivity may, in this context, be associated with better material outcomes for oneself. The paradigm provided here, where participants instruct an AI avatar as to their preferences, which then plays on their behalf, isolates preferences for collaboration while controlling for other cognitive variables and may find fruitful application in the study of relational psychopathology.

## Supplementary Information

Below is the link to the electronic supplementary material.

Supplementary file1 (PDF 246 KB)

## Data Availability

All data used and in the published article, as well as the code used to collect data and prepare this article, is made publicly available at https://github.com/mmoutou/
