# Supplementary material for: When Collaboration Falters, Insensitivity to How Our Actions Affect Others Drives Inflated Self-evaluations
Source: Comput Brain Behav. 2025 Jun 18;9(1):91–103. doi: 10.1007/s42113-025-00250-y (PMC13035595; doi:10.1007/s42113-025-00250-y)
Supplement: Supplementary file 1 — Supplementary file1 (PDF 246 KB) [file 42113_2025_250_MOESM1_ESM.pdf]

Supplement to:

When collaboration falters, insensitivity to how our actions affect others drives inflated self-evaluations

Michael Moutoussis*, Meera Gosalia, Geert-Jan Will, Giles Story, Tobias U. Hauser, Aslinn Bowler, Siobhan Edinboro, the NSPN Consortium, Gita Prabhu, Raymond Dolan

* Corresponding author: m.moutoussis@ucl.ac.uk

Beliefs-about-actions model of self- and other- evaluation

We hypothesized that participants evaluated others depending on the probability that their actions were emitted had they been the 'ideal partner' that was described during instruction of the avatar, here denoted as *g* :

$$p_A(t) = \Pi_{i=1}^{n} p(a_{o,i}; g)^{1/n} \qquad \text{Eq. S1}$$

In these models, we allowed for a magnanimity parameter m, which shifted this probability in a more positive (if m > 1) direction. The report probability, P_A, was mapped to the Likert scale provided for participants, which had n_L levels, by using it as the parameter of a binomial distribution of n_L draws, sharpened (or blunted) by a decision uncertainty parameter *u* :

Eq. S2

$$\begin{aligned} P_A &\propto p_A^{1/m} \\ p(L) &\propto Bin(P_A, n_L)^{1/u} \\ &:= NB(P_A, u, n_L) \end{aligned}$$

We used a 'noisified' binomial distribution, *NB*, to parameterize all unimodal probability mass functions, unless stated otherwise.

To calculate self-approvals through beliefs-about-actions models, a very similar strategy was used. Here, the probabilities forming the product in Eq. S7 were replaced by the probabilities for one's observed actions *under a policy updated in the light of what the partner's decision actually was, based on their now observed actions.* Self-disapproval was then a matter of regret about the actions one took.

Note that the belief-about-actions model of other-evaluation is similar to the eventual model winner – most people would describe an ideal partner as one who was highly collaborative, and hence provide high rewards for the self. However the belief-about-actions model of self-evaluation is not so close to the eventual winner, and indeed did not do well in accounting for

the data.

Details of Reinforcement learning model of self- and other-evaluation

As mentioned in the main text, the full equation for self-esteem update is:

$$\vec{E}^{t} = \vec{E}_0 + \mathbf{W}_x \vec{E}^{t-1} + \mathbf{W}_r \vec{R}^{t} \qquad \text{Eq. S3 [aka Eq. 2 in main text]}$$

The LHS is an *Evaluation vector*, containing self and other approval. The first term of the RHS is the trait-evaluation vector, considered constant. The second term consists of a matrix of weights, which multiplies the previous evaluation. We term this the autoregression weights. The last term of the RHS of this equation (Eq. 2 of main text) turned out to be the most important one. It consisted of the product of a weights matrix with a vector of outcomes for self and other. The weight parameters w_{SS} , w_{SO} , w_{OS} , w_{OS} are described in the main text and Fig. 4. The outcome vector R could, in different versions of the model, contain either returns, or prediction errors, or a weighted sum of the two. In the winning model, it simply contained the returns for self and other. Its parameterized general form was:

$$\vec{R}^{(t)} = (1-\lambda_2)\vec{O}^{(t)} + \lambda_2 \vec{PE}^{(t)} \qquad \text{Eq. S4}$$

The parametrization of the weight terms in terms of Table 1 of the main article has as follows:

$$\mathbf{W_r} = \eta \begin{pmatrix} wOS_{11} & wOS_{12} \\ wOS_{21} & wOS_{22} \end{pmatrix} \qquad \text{Eq. S5}$$

$$\mathbf{W_x} = (1-\eta) \begin{pmatrix} w_{Ex} & 1-w_{Ex} \\ 1-w_{Ex} & w_{Ex} \end{pmatrix} \qquad \text{Eq. S6}$$

In the versions where prediction errors were encoded in R, a further equation was required, that kept track of expected returns V, so that prediction errors could be estimated:

$$\begin{aligned} \vec{V}^{(t+1)} &= \vec{V}^{(t)} + \lambda_1 \vec{PE}^{(t)} \\ \vec{PE}^{(t)} &= \vec{O}^{(t)} - \vec{V}^{(t)} \end{aligned} \qquad \text{Eq. S7}$$

In the limiting case, assuming play has stabilized for a long time so that returns are stably expected and there is no prediction errors, the above can be solved to give:

$$\vec{E}^{\infty} = (I - \mathbf{W}_x)^{-1}(\vec{E}_0 + \mathbf{W}_r \vec{R}^{\infty}) \qquad \text{Eq. S8}$$

Where *I* is the identity matrix. We used this equilibrium expectation, rather than introduce

further parameters, to initiate the Evaluation vector when necessary.

Supplementary results

Note on model fitting and model comparison

In our model fitting, before applying the BIC we used weakly informative priors over parameters, to regularize extreme values yet retain maximum power for further, including exploratory, correlation analyses. For the technically interested reader, these decisions regarding model-fitting and selection, but also the importance of considering the mathematical detail of models to interpret their overlaps, see Moutoussis et al, 2018a https://tinyurl.com/bdexp7k7 and Moutoussis et al, 2018b https://tinyurl.com/2hmrndx7 .

Study 1 person-evaluation results

The key measure used in Study 1 in order to assess the paradigm developed here was the difference in evaluations between self and other. The standard deviation of the difference was 0.97, so it was essentially normalized. The mean difference was 0.76 (Figure S1). We asked the sample size that would be required to detect a Minimum Effect of Interest of half this mean difference, assuming a t distribution. This resulted in N=41. (https://www.gigacalculator.com/calculators/power-sample-size-calculator.php) .

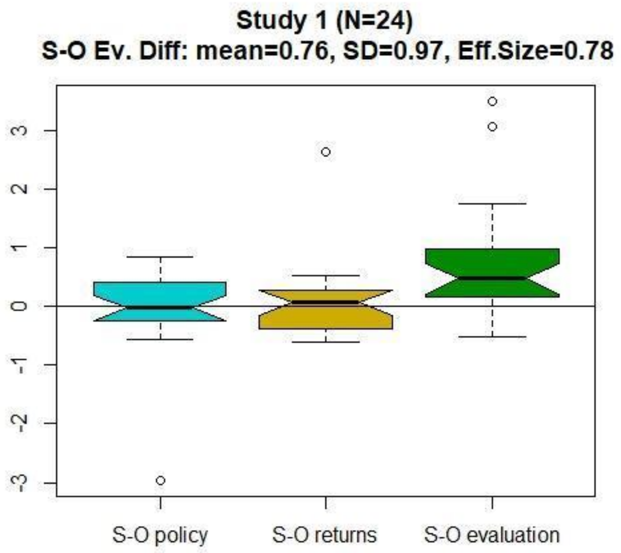

Figure S1 Average decisions, returns and self-serving bias in study 1. Notches estimate the confidence interval of the mean. There was no significant difference in the average decisions that participants and the avatars that they played with made (left, cyan). Similarly, there was no evidence for difference in the average returns (middle, gold). However, there was a significant difference in Self vs. Other average evaluations (right, green), p=0.00086.

Self-positivity synthetic results

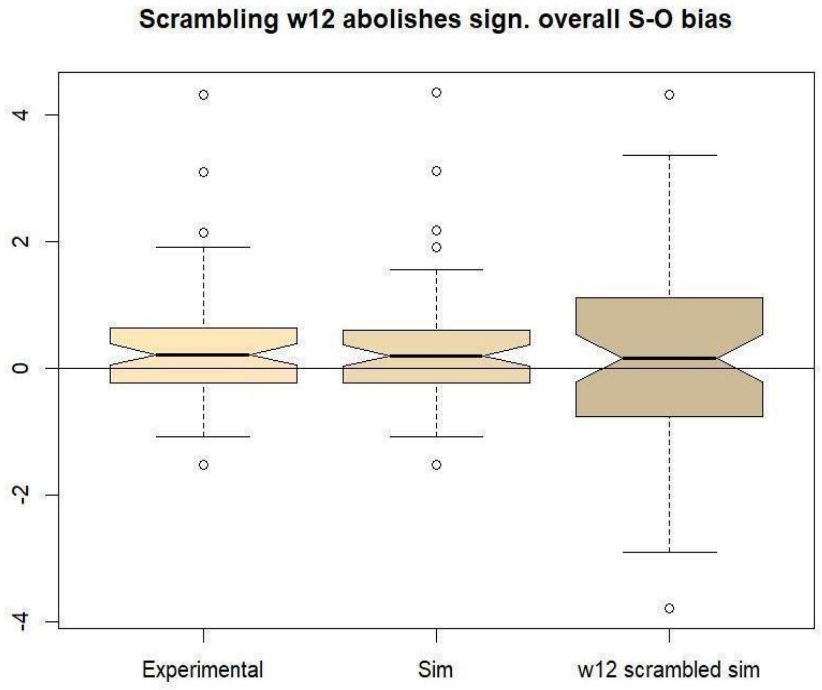

Figure S2 Average self-positivity over the 23 trials for each participant for the experimental data (left-most), synthetic data with best-fit parameters (center) and with the key parameter which is the weight of Other-returns on Self-evaluation scrambled (right). Scrambling these parameters results in no longer statistically significant self-positivity over the sample. Notch=approximate confidence interval for the mean.

Supplemental References

Friston, K. J., Schwartenbeck, P., FitzGerald, T., Moutoussis, M., Behrens, T., & Dolan, R. J. (2013). The anatomy of choice: Active inference and agency. Frontiers Human Neurosci, 7, 1–18. https://doi.org/10.3389/fnhum.2013.00598
